# Developing a Novel Indolocarbazole as Histone Deacetylases Inhibitor against Leukemia Cell Lines

**DOI:** 10.1155/2015/675053

**Published:** 2015-11-16

**Authors:** Wenjing Wang, Maomin Lv, Xiong Zhao, Jingang Zhang

**Affiliations:** Department of Blood Biopharmaceuticals and Viral Detection, Institute of Transfusion Medicine, The Academy of Military Medical Sciences, Beijing 100850, China

## Abstract

A novel indolocarbazole (named as ZW2-1) possessing HDAC inhibition activity was synthesized and evaluated against human leukemia cell lines HL-60 and NB4. ZW2-1 performed anti-population growth effect which was in a concentration-dependent manner (2–12 *μ*M) by inducing both apoptosis and autophagy in cells. The compound also caused differentiation of HL-60 and NB4 cells as shown by increasing expression of CD11b, CD14, and CD38 at moderate concentration (4 *μ*M). At relatively high concentration (8 *μ*M), ZW2-1 significantly decreased intracellular histone deacetylase 1 level which was also observed. All the results indicated that ZW2-1 could be a novel antileukemia lead capable of simultaneously inducing apoptosis, autophagy, and differentiation.

## 1. Introduction

Leukemia is one of the most common malignancies worldwide [1]. Blocking cells differentiation at early stage and inability of cells to differentiate into functional mature cells are the main characteristics of leukemia; this causes bone marrow accumulation of the leukemic cells and eventually leukemization and organ infiltration [2, 3]. Although a therapy based on the induction of differentiation such as using all-transretinoic acid (ATRA) has favorable outcomes [4], it has been limited by causing progressive resistance and a number of side effects [5–7]. Thus, the development of novel antileukemia agents attracts a large amount of interest [8]. One potential class of therapeutic agents for leukemia is histone deacetylase (HDAC) inhibitors [9].

Histone deacetylases (HDAC) are a family of enzymes playing a crucial role in chromatin remodeling therefore affecting transcriptional processes [10]. Aberrant activity of HDAC has been found in several human cancers including leukemia [11, 12]. As clinically validated cancer targets, their inhibition has been proven to be successful strategy for the development of novel anticancer agents. HDAC inhibitors (HDACi) mediate cancer cell death through several pathways and are able to induce apoptosis, differentiation, cells cycle arrest, inhibition of DNA repair, upregulation or reactivation of silenced tumor suppressors, downregulation of growth factors, autophagy, and control of angiogenesis [13–15]. Notably, preclinical and clinical studies of HDAC inhibitors conducted in leukemia have shown potent anticancer effects [16, 17].

Indole alkaloids constitute a group of natural products that have attracted great attention as anticancer leading compounds [18, 19]. As a unique class of indole alkaloids, indolocarbazoles had been reported with an array of interesting biological activities [20]. The most significant biological profile of these compounds is their potential antitumor effects and the activity may be due to different mechanisms of action, including DNA intercalation, inhibition of DNA topoisomerases, and inhibition of protein kinases [21]. Great efforts are made to generate indolocarbazole derivatives with improved properties for the treatment of cancer [22]. Various biological activities have been studied for indolocarbazoles, but rarely as HDAC inhibitor.

**Figure 1:**
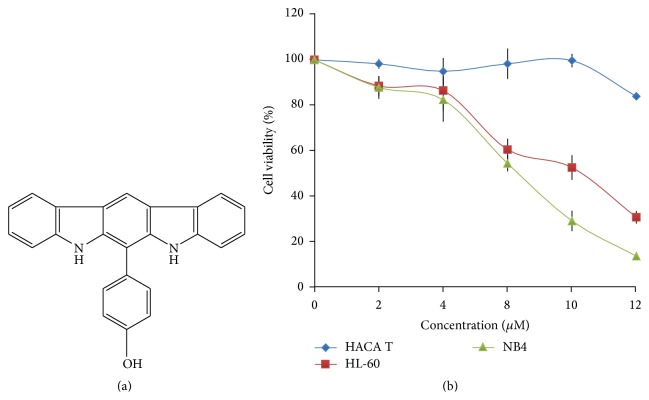
Structure of ZW2-1 and its effects on cell proliferation. (a) Chemical structure of ZW2-1. (b) Effects of ZW2-1 on cell proliferation of human leukemia cell line HL-60, NB4, and immortal human keratinocyte cell line Haca T. The values are represented as mean ± SD of three independent experiments with five replicates in each.

In this paper a novel antileukemia agent, 4-(5,7-dihydroindolo[2,3-b]carbazol-6-yl)phenol (named as ZW2-1, Figure 1(a)), possessing potential HDAC inhibition activity was reported. ZW2-1 was prepared via the chemical synthesis method described in Supporting Information in Supplementary Material available online at http://dx.doi.org/10.1155/2015/675053. The synthesized product was purified by HPLC with a purity of 98.9% and analyzed using NMR and MS, and data were also provided in Supporting Information.

## 2. Materials and Methods

### 2.1. Cell Lines and Cell Culture

HL-60 (human myeloblastic leukemia cells line), NB4 (human acute promyelocytic leukemia cell line), and HACA T (human keratinocyte cell) were kindly provided by Professor Ming Zhao (College of Pharmaceutical Sciences, Capital Medical University, Beijing, China). HL-60 and NB4 were cultured in RPMI-1640 medium with 10% fetal bovine serum and 1% (v/v) penicillin-streptomycin (10000 U/mL) in 5% CO_2_ at 37°C, and HACA T cells were cultured in DMEM : F12 (1 : 1) medium with 10% fetal bovine serum and 1% (v/v) penicillin-streptomycin (10000 U/mL) in 5% CO_2_ at 37°C. The cytotoxicity of the compound ZW2-1 was assessed using a cell proliferation assay developed by Promega (CellTiter 96 AQueous one solution cell proliferation assay).

### 2.2. Cell Viability Assay

Cells were plated in triplicate wells in 96-well plates (4 × 10^3^ cells/well) cultured overnight followed by exposing to different concentrations of ZW2-1 (0, 2, 4, 8, 10, and 12 *μ*M), and medium with same concentrations of DMSO was used as control. The cell proliferation was monitored at 48 h using the MTS assay according to the instructions, and the absorbance was read at 490 nm using a SpectraMax M5 Microplate Reader (Molecular Devices Instruments Inc., USA).

### 2.3. Determination of Apoptosis

Cells were seeded into 6-well plates at 5 × 10^5^ cells/well. After overnight incubation, they were treated with 8 *μ*M DIM for 48 h and medium with same concentrations of DMSO was used as control. After incubation, cells were harvested, then double stained with Annexin V-FITC/PI using an apoptosis analysis kit (KeyGEN BioTECH, CHN), and subjected to flow cytometry analysis for detection of apoptosis. 10,000 cells per sample were analyzed by a BD FACSCalibur Cytometry (BD Biosciences) to quantify apoptotic cells (Annexin V-FITC positive cells).

### 2.4. Analysis of Mitochondrial Transmembrane Potential

One of the hallmarks of apoptosis is mitochondrial disruption, which is characterized by changes in the mitochondrial membrane potential. In our study, the mitochondrial transmembrane electrochemical gradient was measured using JC-1 (Invitrogen, Carlsbad, CA, USA). As a cell permeable lipophilic dye, JC-1 has the ability of freely crossing the mitochondrial membrane and forming J-aggregates which fluoresce red; accordingly, untreated cells with a normal mitochondrial membrane potential when stained with JC-1 exhibit a pronounced red fluorescence (PE). After an apoptotic stimulus, the resultant decrease in the mitochondrial membrane potential prevents JC-1 from entering the mitochondria and remains as monomers in the cytosol that emits a green fluorescence (FITC). Therefore, the ratio of J-aggregates/monomers serves as an effective indicator of the cellular mitochondrial transmembrane potential, allowing apoptotic cells to be easily distinguished from their nonapoptotic counterparts. Briefly, HL-60 and NB4 cells were incubated with ZW2-1 for 48 hr, and cells (1 × 10^6^/mL) were then incubated with JC-1 (10 mM) for 30 min and washed with PBS. Both red and green fluorescence emissions were analyzed by flow cytometry (BD, FACSCalibur, USA) using an excitation wavelength of 488 nm and observation wavelengths of 530 nm for green fluorescence and 585 nm for red fluorescence.

### 2.5. Evaluation of the Surface Markers CD38, CD14, and CD11b

The expression of cell surface differentiation markers was quantified using flow cytometry. Cells were treated with ZW2-1 (4 *μ*M), and medium with same concentrations of DMSO was used as control. The cells were then washed twice with cold PBS with 0.09% sodium azide and 1% (v/w) bovine serum albumin (BSA) and incubated on ice with antibody conjugated with fluorescein isothiocyanate (FITC conjugated CD11b, FITC conjugated CD14, and PE conjugated CD38; all from Biolegend, Inc., San Diego, CA, USA) in the proportion of 1 : 20, for 30 min. A total of 10,000 cells were analyzed by flow cytometry (FACSCalibur, BD, USA) and the frequency of CD11b-positive, CD14-positive, and CD38-positive cells was determined by using a Flowjo 7.6.1 software.

### 2.6. The TEM Observation

To detect whether autophagy was induced in ZW2-1 treated HL-60 cells, transmission electron microscopy (TEM) was applied. After HL-60 cells were incubated for 48 h with ZW2-1 (8 *μ*M), the cells were washed with PBS and then centrifuged at 1500 rpm for 10 min. The supernatants were removed. The cell pellets were fixed in a 0.1 M PBS solution containing 2.5% glutaraldehyde for 2 h. They were then washed with 0.1 M PBS, embedded in 2% agarose gel, postfixed in 4% osmium tetroxide solution for 1 h, washed with distilled water, stained with 0.5% uranyl acetate for 1 h, dehydrated in a graded series of ethanol (30%, 60%, 70%, 90%, and 100%), and embedded in epoxy resin. The resin was polymerized at 60°C for 48 h. Ultrathin sections obtained with ultramicrotome were stained with 5% aqueous uranyl acetate and 2% aqueous lead citrate, air-dried, and imaged under a transmission electron microscope (TEM) (JEOL JEM2100, Japan).

### 2.7. Western Blot Analysis

HL-60 cells were seeded into 6-well plates at 5 × 10^5^ cells/well. After overnight incubation, they were pretreated with 8 *μ*M ZW2-1 for 48 h. Cells were then harvested and washed with ice-cold PBS, lysed with ice-cold RIPA lysis buffer (KeyGEN BioTECH, CHN) with 1 mmol/L PMSF. Protein concentrations were calculated by BCA assay kits (Thermo Fisher SCIENTIFIC, CHN). 20 *μ*g of total cellular protein was subjected to 12% SDS-PAGE and transferred to PVDF membranes (Millipore, Atlanta, GA, USA). The membranes were blocked with 5% defatted milk powder at room temperature for 1 hr and then immunoblotting was performed with primary antibodies at 4°C overnight, followed by HRP-conjugated secondary antibody at room temperature for 1 hr. Following each step, the membranes were washed five times with PBS-T for 5 min. Finally, the blots were developed using the enhanced chemiluminescence (ECL) system (Pierce Chemical, 34080).

### 2.8. HDAC Activity Assay

Histone deacetylases are a class of enzymes that remove the acetyl groups from the lysine residues leading to the formation of a condensed and transcriptionally silenced chromatin. The protein plays an important role in the control of cell proliferation and differentiation. To assess whether ZW2-1 was able to inhibit HDAC1 in HL-60 and NB4 cells, a colorimetric sandwich ELISA kit (Proteintech, USA) was used to detect and quantify protein levels of endogenous HDAC1. Cells were seeded into 6-well plates at 5 × 10^5^ cells/well. After overnight incubation, they were treated with 8 *μ*M ZW2-1 for 24, 48, and 72 h. Cells were then harvested and washed with ice-cold PBS, lysed with ice-cold RIPA lysis buffer with 1 mmol/L PMSF. Protein concentrations were calculated by BCA assay kits (Thermo Fisher SCIENTIFIC, Beijing, China), and 50 *μ*g of total cellular protein of each sample was plated in triplicate wells. HDAC1 activity was measured with the corresponding detection kit according to the manufacturer's instructions.

## 3. Results

### 3.1. ZW2-1 Inhibits HL-60 Cell Proliferation

The compound ZW2-1 inhibiting leukemia cell proliferation was determined by MTS assay in HL-60, NB4, and Haca T cells. The inhibitions are shown in Figure 1(b), as the concentration curves demonstrated that ZW2-1 blocks HL-60 cell and NB4 cell proliferation in a concentration-dependent manner. The viabilities of HL-60 cells treated with 4, 8, and 12 *μ*M of ZW2-1 for 48 hr were 86.6%, 60.9%, and 31.4%, respectively, and were 82.6%, 54.8%, and 14.5% for NB4 cells, respectively, but were 95%, 98.3%, and 84.1% in the case of Haca T cells, respectively. The results indicated that in contrast to HL-60 and NB4 cells ZW2-1 displayed a nonsignificant cytotoxic effect on Haca T cells.

### 3.2. ZW2-1 Induces Apoptosis in HL-60 Cells

**Figure 2:**
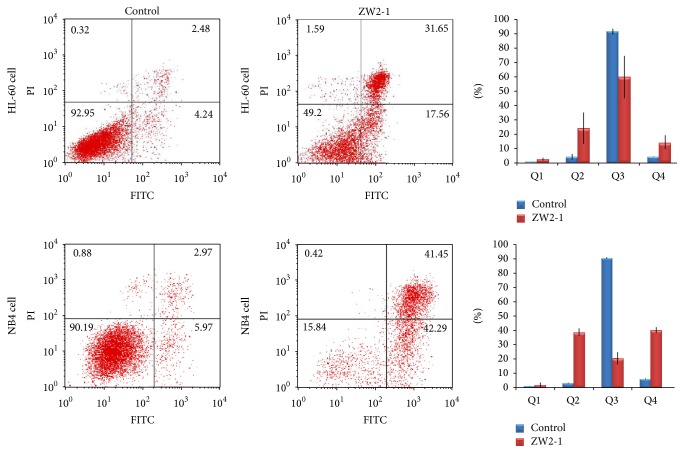
Effects of ZW2-1 on apoptosis of HL-60 and NB4 cells. Annexin V and PI double staining for apoptosis of cells 48 h after incubation with ZW2-1 in comparison to the untreated control cells. All experiments are presented as mean ± SD of at least three independent experiments performed in replicates.

To elucidate the possible mechanism(s) of inhibiting proliferation of HL-60 cells, we have tested the effects of ZW2-1 to induce apoptosis in HL-60 and NB4 cells by flow cytometric analysis of Annexin V/FITC and propidium iodide (PI) uptake. As shown in Figure 2, treatment with 8 *μ*M ZW2-1 for 48 hr resulted in apoptotic cell death in HL-60 cells (49.2%) and NB4 cells (78.3%). The results indicated that cytotoxicity of ZW2-1 (8 *μ*M, 48 hr) in HL-60 and NB4, at least partly, resulted from apoptosis.

### 3.3. ZW2-1 Induces Mitochondria-Mediated Apoptosis in HL-60 Cells

**Figure 3:**
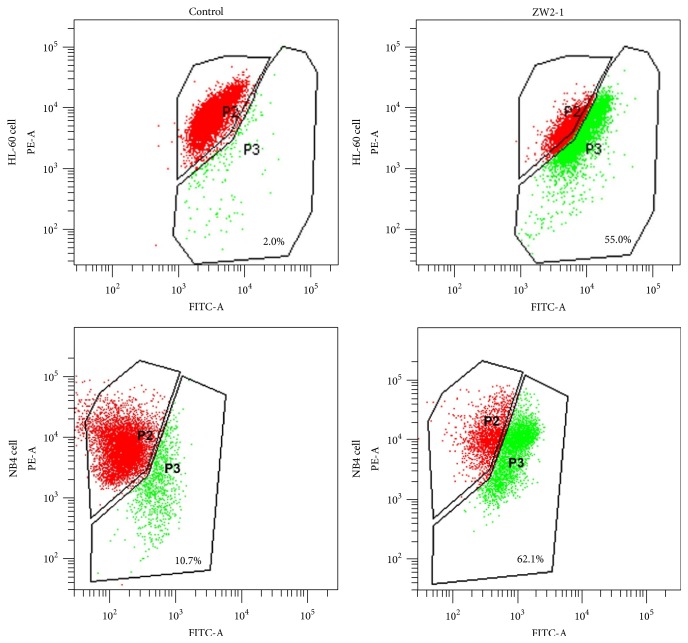
Effects of ZW2-1 on mitochondria membrane electrochemical potential of HL-60 cells. HL-60 cells were stained with JC-1 dye (P2: aggregated JC-1, red fluorescence (PE); P3: monomeric JC-1, green fluorescence (FITC)) which was measured by flow cytometry. The value represents the average percentage of cells in each gate (*n* = 3).

Loss of the mitochondrial membrane potential (MMP) is a hallmark of intrinsic apoptosis, because it is associated with the release of proapoptotic proteins into the cytosol. To assess mitochondrial membrane potential, HL-60 and NB4 cells were incubated with ZW2-1 for 48 hr, and cells were then incubated with JC-1 and were analyzed by both red and green fluorescence emissions by flow cytometry. After treatment with 8 *μ*M ZW2-1 for 48 hr, the proportion of the cells having mitochondrial membrane dysfunction increased from 2.0% to 55.0% in HL-60 cells and from 10.7% to 62.1% in NB4 cells, suggesting that ZW2-1 treatment resulted in a loss of mitochondrial membrane potential in AML cells (Figure 3).

### 3.4. ZW2-1 Induces HL-60 Cell Autophagy

**Figure 4:**
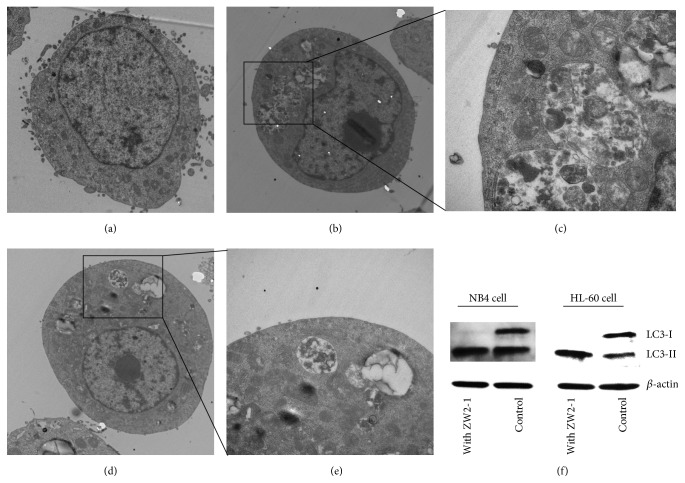
TEM images of HL-60 cells treated with ZW2-1 and western blots analysis of autophagy marker protein LC3. (a) Control group. (b, d) Cells were exposed to ZW2-1. (c, e) The magnification of selected area. (f) Effect of ZW2-1 on the LC3-I/LC3-II conversion.

In order to observe the activation of autophagy of ZW2-1 (8 *μ*M, 48 hr) treated HL-60 cells, the TEM ultrastructural analysis was performed. The autophagic ultrastructural features are shown in Figure 4. The typical autophagic vacuoles (Figure 4(e)), three obviously larger autophagic vacuoles, contained partially degraded cytoplasmic materials (Figure 4(c)), and the control cells (Figure 4(a)) are compared. The ZW2-1 induced autophagy was further verified by assessing the LC3-I/LC3-II conversion. The western blot analysis showed that the LC3-II/LC3-I ratio was significantly elevated, indicating that the autophagic activity was enhanced by ZW2-1 (Figure 4(f)), and ZW2-1 induces autophagy as well as apoptosis.

### 3.5. ZW2-1 Promotes Differentiation in HL-60 Cells

To determine differentiation of HL-60 and NB4 cells induced by ZW2-1, fluorescence activated cell-sorting (FACS) was carried out to monitor three surface proteins (CD11b, CD14, and CD38), characteristic of differentiated HL-60 and NB4 cells.

**Figure 5:**
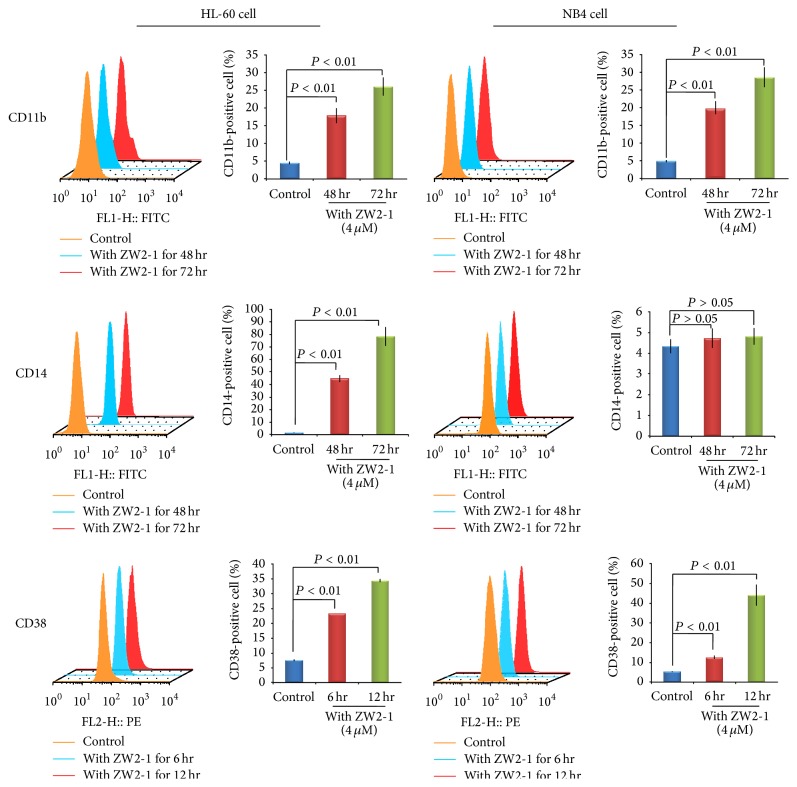
Effects of ZW2-1 on CD11b, CD14, and CD38 expression. Flow cytometric analysis of expression of CD11b and CD14 at 0, 48, and 72 hr after treatment with ZW2-1, respectively, and expression of CD38 at 0, 6, and 12 hr after treatment with ZW2-1. To measure the percent positive signal, control group was set to exclude about 95% of the live cell population peak. All experiments are presented as mean ± SD of three independent experiments performed in replicates.

Since CD38 is one of the earliest markers of progressive differentiation, we investigated the CD38 expression after 0, 6, and 12 hr incubation with ZW2-1. FACS analysis revealed that ZW2-1 treatment significantly increased CD38 expression both in HL-60 (from 5.13% to 12.5% and 44.1%) and in NB4 (from 7.39% to 23.2% and 34.4%) cell (Figure 5).

After 48 and 72 hr incubation with ZW2-1, both CD11b and CD14 expressions were increased in HL-60 (from 4.6% to 8.4% and 22% of CD11b, resp.; from 1.57% to 44.9% and 78.7% of CD14, resp.); in the case of NB4 cells, only CD11b expression was significantly increased (from 4.9% to 19.6% and 28.6%, resp.), and CD14 expression levels were not significantly different compared to control cells, suggesting that ZW2-1 does not affect CD14 expression in NB4 cell (Figure 5).

### 3.6. ZW2-1 Administration Decreased HDAC1 in HL-60 Cells

**Figure 6:**
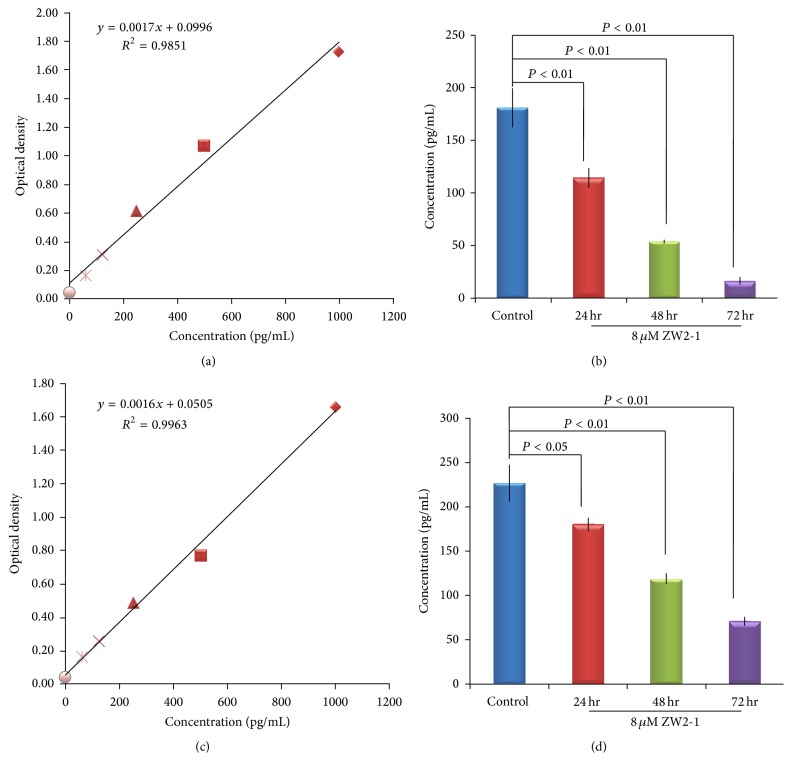
Effects of ZW2-1 on histone acetylation 1 in HL-60 cells. Colorimetric sandwich ELISA assay was used to detect the HDAC1 level in HL-60 and NB4 cells after treatment with ZW2-1 for 0, 24, 48, and 72 h. (a) Standard curve for samples of HL-60 cells. (b) HDAC1 concentrations of HL-60 cells treated with ZW2-1. (c) Standard curve for samples of NB4 cells. (d) HDAC1 concentrations of NB4 cells treated with ZW2-1. Data represents the means of three repeats ± SD.

To determine the HDAC1 inhibition activity of ZW2-1, we tested the HDAC1 levels in HL-60 and NB4 cells before and after treatment with ZW2-1 by ELISA (Figure 6). HL-60 and NB4 cells were treated with 8 *μ*M ZW2-1 and HDAC1 enzymatic activities were measured after for 24, 48, and 72 hours. As the results showed, intracellular concentration of HDAC was reduced by 36.9% (*P* < 0.01), 70% (*P* < 0.01), and 90.8% (*P* < 0.01) in HL-60 cells and by 20.6% (*P* < 0.05), 47.3% (*P* < 0.01), and 68.6% (*P* < 0.01) in NB4 cells following exposure to 8 *μ*M ZW2-1 for 24, 48, and 72 hours, respectively.

## 4. Discussion

Histone deacetylase inhibitor(s) (HDACi) are epigenetic drugs with ability to promote cellular differentiation, senescence, and apoptosis. In recent years, increasing numbers of researchers have embarked on the development of novel small molecules that are possessing histone deacetylase inhibition activity as potent antileukemia agents [23, 24].

In the present study, we investigated the cytotoxicity effects of a novel indolocarbazole ZW2-1 on HL-60 and NB4 leukemia cells and detected its autophagy and apoptosis-inducing effects at cell levels, so as to illuminate the possible mechanisms involved in ZW2-1-caused cell death.

To test the cell viability after exposure to ZW2-1, we applied two types of cells, HL-60 and NB4 (human leukemia cell line) and HACA T (immortal human keratinocyte cell line). According to our results, ZW2-1 can effectively block both HL-60 and NB4 cells proliferation but displayed nonsignificant cytotoxic effect on Haca T cells at the same concentration.

Analyzing the killing process of ZW2-1 we observed apoptosis-related mechanisms with various experimental approaches. Apoptosis evaluation based on Annexin V/PI double-staining assay showed a remarkably increased percentage of apoptotic cells in ZW2-1 treated group compared to blank control. We also assessed the loss of mitochondrial membrane potential caused by ZW2-1 using JC-1 staining assay. Index of green fluorescence (JC-1 monomers) which is considered an exceptionally specific marker for apoptosis is significantly increased in ZW2-1 treated group compared with control.

ZW2-1 induced autophagy was tested using both TEM observation and western blot assay for LC3-I/LC3-II conversion. Typical autophagosomes were viewed in ZW2-1 treated group compared with the normal sample, and the LC3-II/LC3-I ratio was significantly elevated after incubation with ZW2-1. The results indicated an autophagy induction activity of ZW2-1.

As for cells differentiation, we measured the cell surface markers CD11b, CD14, and CD38 by FACS analysis. The percentages of CD38- and CD11b-positive cells were significantly increased in both HL-60 and NB4 cells induced by 4 *μ*M ZW2-1; however, CD14 expression was only induced in HL-60 cells.

Previous study indicated that aberrant expression of HDAC1 appears common in tumors including leukemia and is associated with enhanced proliferation and defect in autophagy [25]. From the data presented, it appears that HDAC1 inhibition activity of ZW2-1 is well correlating with induction of apoptosis, autophagy, cell differentiation, and cell growth arrest. Therefore, ZW2-1 could be a promising candidate as antileukemia agent. Thus, our findings may provide a new scientific insight into differentiation induction and may suggest a novel strategy model for leukemia therapy.

## Supplementary Material

Chemical synthesis method and structure information of compound ZW2-1.
